# Detection and Diagnostic Accuracy of Cardiac Arrhythmias Using Wearable Health Devices: A Systematic Review

**DOI:** 10.7759/cureus.50952

**Published:** 2023-12-22

**Authors:** Hadrian Hoang-Vu Tran, Neway A Urgessa, Prabhitha Geethakumari, Prathima Kampa, Rakesh Parchuri, Renu Bhandari, Ali R Alnasser, Aqsa Akram, Saikat Kar, Fatema Osman, Ghadi D Mashat, Lubna Mohammed

**Affiliations:** 1 Internal Medicine, California Institute of Behavioral Neurosciences & Psychology, Fairfield, USA; 2 Research, California Institute of Behavioral Neurosciences & Psychology, Fairfield, USA; 3 Internal Medicine, California Institute of Behavioural Neurosciences & Psycholgy, Fairfield, USA; 4 Internal Medicine, Manipal College of Medical Sciences, Pokhara, NPL; 5 Internal Medicine/Family Medicine, California Institute of Behavioral Neurosciences & Psychology, Fairfield, USA; 6 General Surgery, California Institute of Behavioral Neurosciences & Psychology, Fairfield, USA; 7 Internal Medicine, Dallah Hospital, Riyadh, SAU; 8 Neurosciences and Psychology, California Institute of Behavioral Neurosciences & Psychology, Fairfield, USA; 9 Pediatrics, California Institute of Behavioral Neurosciences & Psychology, Fairfield, USA

**Keywords:** atrial fibrillation (af), photoplethysmography, stroke, wearable device, cardiac arrhythmias (ca)

## Abstract

Photoplethysmography (PPG) is the wearable devices' most widely used technology for monitoring heart rate. The systematic review used the Preferred Reporting Items for Systematic Reviews and Meta-Analyses (PRISMA) standards and guidelines. This systematic review seeks to establish the effects of wearable health devices on cardiac arrhythmias concerning their impact on the personalization of cardiac management, their refining effect on stroke prevention strategies, and their influence on research and preventive care of cardiac arrhythmias and their re-evaluation of the patient-physician relationship. The population, exposure, control, outcomes, and studies (PECOS) criteria were used in the systematic review. This review considered studies that covered the tests conducted on individuals who presented with cardiovascular diseases (CVD) and also healthy people. The intervention for studies included wearable health devices that could detect and diagnose cardiac arrhythmias.

The study considered articles that reported on the personalization of cardiac management, stroke prevention strategies, influence in research and preventive care of cardiac arrhythmias, and the re-evaluation of the patient-physician relationship. Two independent researchers were used in the extraction of the data. In case of dispute, the issue was resolved using a third party. The study's quality analysis was conducted using AXIS. The management of atrial fibrillation (AF) lies heavily in the prevention of stroke. The accuracy being reported in the prediction of arrhythmias and the monitoring of heart rates makes wearable devices an efficient means to personalize health care. Personalization of health and treatment in preventing and managing arrhythmias becomes possible due to the portability of smart wearable devices. However, limitations may be observed due to the high costs incurred in their purchase and use. Using smart wearable devices for the detection of cardiac arrhythmias was very significant.

## Introduction and background

Technological advancements have transformed the way humans approach healthcare to a huge degree. The menace of cardiovascular diseases (CVD) and the global burden of the same have necessitated new inventions for monitoring, preventing, and treating such health issues. There has been an exponential growth of wearable devices for tracking and health purposes [1]. Wearable devices included electronic shirts, glasses, wristbands, and in-ear monitors. Each device, depending on the type and company, varied in its capacities to monitor physical activity, heart rhythm, sleep patterns, heart rate, respiratory rate, and blood pressure. Photoplethysmography (PPG) was the most widely used technology for monitoring the heart rate in wearable devices [1]. Electrocardiograph information cannot be measured using PPG, which limits the noise detection and the accuracy of wearable devices. PPG technology has proved nearly 93% accurate in detecting life-threatening cardiac arrhythmias. These devices have been noted to help in the detection of cardiac arrhythmias. Wearable devices bring in a new-found perspective on the healthcare sector. From detection to monitoring to preventing cardiac arrhythmias, they have been identified to either help or be disruptive to the healthcare system.

Patients and physicians have been receptive to these wearables' safety and assurance. Wearable devices in the health sector allow continuous monitoring of a patient's vital signs [2]. Such development allowed for an increase in safety for the patients. According to the Food and Drug Administration (FDA) standards, Wearable health devices should be unobtrusive and reliable [2]. In addition, according to the FDA standards, the false alarm rate should be low to avoid alarm fatigue. Alarm fatigue has been associated with desensitizing patients and health professionals to the alarms, especially if they are many and false [3]. 

Cardiovascular concerns are predicted by various factors that spread across an individual's lifestyle and demographic traits. The increase in age raises health concerns for atrial fibrillation (AF) as a global epidemic [3]. Wearable devices that are made to monitor electrocardiographic (ECG) signals and heart rate are important in the detection of arrhythmias. Artificial intelligence and machine learning have been identified as critical components for the optimization and effectiveness of wearable devices. Wearable devices enhance the management and prevention of CVD such as cardiac arrhythmias [3]. This results from continuous real-time monitoring of an individual's accurate health conditions. AF is the most sustained arrhythmia, with a risk of 37% for individuals 55 years and above [4]. AF has been known to increase by fivefold the risk of stroke among patients clinically and has been recorded to account for a third of all strokes. It has been reported that 20% of the cases of stroke caused by AF had diagnosis and detection made after the stroke [4]. Early detection of AF leading to therapeutic anticoagulation helps in a 2.7% reduction of the absolute risk of strokes and 8.4% in secondary prevention every year [5]. The screening of AF routinely is recommended during medical visits to help reduce the risks [6]. Intermittent and sustained arrhythmias are among the conditions that mark CVD [7]. In the study, it is further noted that arrhythmias may reflect underlying cardiac problems such as fibrosis, inflammation, myocardial tissue inhomogeneity, and ischemia [7]. As a result, other studies have linked AF to increased re-hospitalization, mortality, and sudden cardiac arrests [8,9].

Therefore, detecting arrhythmias in the early stages becomes crucial in avoiding catastrophic and worsened situations. Early detection allows for the patients to be attended to promptly. Arrhythmia is described as rhythm disturbances among individuals who are symptomatic or not [7]. Wearable cardioverter defibrillators increasingly help protect patients during vulnerable times [10]. Its use comes in handy after the diagnosis of heart-related conditions. Ambulatory monitoring devices have become important in collecting and analyzing long-term data that can be used in diagnosing patients [11]. Digitalization and miniaturization of health devices have become very important in the approach toward "4P medicine" [12]. These include prediction, precision, prevention, and personalization. Significant technologies have been made within the past decade [13]. Other studies identify medical techs using blood pressure monitors, mobile phone apps, and single-lead cardiac event monitors [14]. These technological appliances make use of photoplethysmographic signals to monitor the person. PPG technology has been reported to be promising in screening AF as it easily measures the change in blood volume through the skin capillary bed [15]. PPG technology is used on most smart bands available and in heart rate sensors [16]. The use of easily available devices increases the chances of detecting asymptomatic AF. Wearable health devices have increasingly been used in monitoring cardiac diseases. Therefore, it is necessary to understand their accuracy in detecting and diagnosing cardiac arrhythmias.

Aims and objectives

This systematic review seeks to establish the effects of wearable health devices on cardiac arrhythmias concerning their impact on the personalization of cardiac management, their refining effect on stroke prevention strategies, and their influence on research and preventive care of cardiac arrhythmias and their re-evaluation of the patient-physician relationship.

## Review

Methods

Study Design

This systematic review used the Preferred Reporting Items for Systematic Reviews and Meta-Analyses (PRISMA) standards and guidelines. In addition, published PRISMA extension from the Cochrane Handbook for Systematic Reviews and Extensions - Chapter 4 was used to prepare this systematic review [17].

Search Strategy

The systematic review used PubMed, Web of Science, Medline, and Embase as electronic databases to search relevant articles and journals. The search utilized keyword combinations, Boolean operators "AND" and "OR," truncations, and field tags. The medical subject headings (Mesh) was also used in the identification of articles that were tagged and were found relevant to the study. For an accurate and easier search strategy, the named elements of the search were used to build the search strings. Google Scholar was also used to supplement the available materials and data for being a reliable reservoir for gray literature. Studies identified were sought keenly to get the best and most necessary for the systematic review. The search was executed in August 2022. Table 1 shows the MeSH terms and keywords used in the search strategy in this study.

**Table 1:** Search strategy used in the study. MeSH, medical subject heading.

Database	Search Strategy
PubMed	"Wearable Electronic Device*"[Mesh], "Wearable Health Devices" [tw], "Cardiac Arrhythmia" [tw], "Arrhythmias, Cardiac"[Mesh], "Arrhythmia" [tw]
MedLine	"Atrial Fibrillation" [Mesh], "Wearable device*" [tw], "Cardiac Arrhythmia" [tw], "Arrhythmias, Cardiac"[Mesh], "Arrhythmia" [tw]
Web of Science	Atrial Fibrillation, Cardiac Arrhythmia, Wearable Health Devices, Detecting and Diagnosis, Accuracy
Embase	Cardiac Arrhythmia, Wearable Electronic Device, Atrial Fibrillation, Accuracy, Detection and Diagnosis

Eligibility Criteria

The researchers conducting this systematic review agreed on the selected eligibility guidelines for the studies. The population, exposure, control, outcomes, and studies (PECOS) criteria were used in the selection of journals. The population considered was on studies that covered the tests conducted on individuals who presented with CVDs and healthy people as well. The exposure considered for studies included the use of wearable health devices that could detect and diagnose cardiac arrhythmias. On control, this systematic review accepted studies conducted without the presence of a specific experimental comparator. Outcome for the study considered articles that reported on the personalization of cardiac management, stroke prevention strategies, influence in research and preventive care of cardiac arrhythmias, and the re-evaluation of the patient-physician relationship. With regard to study design, the study highly prioritized randomized control trial studies and clinical trials. However, articles with important data were also considered for the systematic review. The researchers only included studies that were published or translated into English.

Quality Assessment

A quality appraisal for the selected studies was performed using AXIS criteria. AXIS critical appraisal entails assessing the degree of bias present in the analysis as well as the methodological quality of the study design. The goal is to methodically evaluate scientific research findings in order to determine their trustworthiness, value, and relevance in a particular context. The tool incorporates aspects related to the quality of reporting, study design, and the possible introduction of biases in the study [18]. The tool consists of 20 questions, with seven questions related to the quality of reporting, seven related to study design quality, and six related to the possible introduction of biases in the study. The quality assessment sections were divided into four. The first section was on the introduction and its validity on the aims and objectives of the study. The second section was on the validity of the methods for the included studies. The third and fourth sections covered the results and discussions, respectively. Answers for the assessment questions were categorized into yes, no, and not applicable, which were abbreviated to "Y," "N," and "NA," respectively.

Data Extraction

Two independent researchers were used in the extraction of the data. The researchers used a pre-designed Excel worksheet in the extraction of relevant data. First, they extracted the authors, year of publication, and demographics from the selected articles. They then extracted the aim, outcomes, results, and conclusions of the selected articles. The researchers then analyzed the collected data to ensure harmony. In case of dispute, the issue was resolved using a third party.

Results Analysis

A single form of investigative analysis was used in this systematic review. A qualitative assessment, a systematic review, was used in this study. The systematic review was conducted using a literal analysis from the included studies.

Results

Study Selection

**Figure 1:**
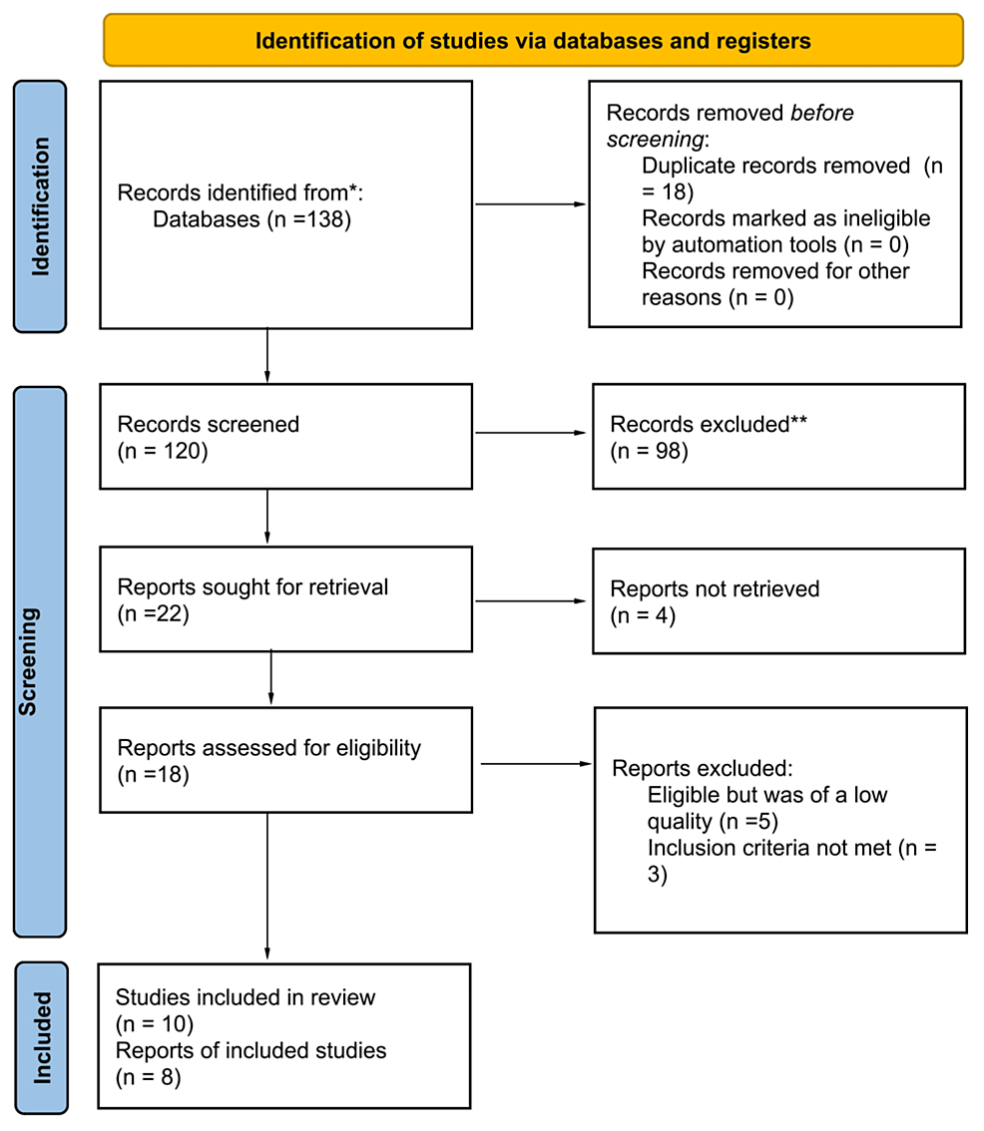
PRISMA flow diagram of the inclusion and exclusion process of the studies in the systematic review. n, number of records; PRISMA, Preferred Reporting Items for Systematic Reviews and Meta-Analyses.

An extensive search study was conducted using PubMed, Web of Science, Medline, and Embase as electronic databases. Using the search strings created, 138 articles were identified. Eighteen duplicate copies were excluded from the records before any screening was conducted. From the 120 remaining articles, screening was conducted on their titles, abstracts, and whether they were full articles. Ninety-eight journals were excluded for not achieving the set standards. Twenty-two journals sought for retrieval were identified, while four were not retrieved and hence their exclusion. From the 18 articles assessed in depth for eligibility, five were excluded for being of low quality, and three were excluded for not meeting the inclusion criteria. A total of 10 studies were included for a systematic review. Figure 1 represents a PRISMA flow chart diagram summarizing the study selection process [1,3,4,11,19-24].

Quality Analysis

AXIS was used to conduct the quality assessment of the 10 studies included in this systematic review. The analysis was done on the validity of a study's four sections, including the introduction, methods, results, and discussion. Table 2 shows the results of the quality appraisal for the included studies [1,3,4,10,11,19,20,22-24].

**Table 2:** AXIS Assessment appraisal results of the studies included. Y, yes; N, no; NA, not applicable.

	Bumgarner et al. (2018) [19]	Cheung et al. (2018) [1]	Dagher et al. (2020) [3]	Nelson and Allen (2019) [20]	Prasitlumkum et al. (2021) [24]	Sana et al. (2020) [11]	Steinhubl et al. (2018) [4]	Tscholl et al. (2021) [10]	Wasserlauf et al. (2019) [22]	Zhang et al. (2014) [23]
Introduction										
Were clear aims and objectives of the study used?	Y	Y	Y	Y	Y	Y	Y	Y	Y	Y
Methods										
Was the study design appropriate for the stated aim of the study?	Y	Y	Y	Y	Y	Y	Y	Y	Y	Y
Was the sample size simplified?	Y	NA	NA	Y	Y	Y	Y	Y	Y	Y
Was the target/reference population clearly defined? (Is it clear who the research was about?)	Y	NA	NA	Y	Y	Y	Y	Y	Y	Y
Was the sample frame taken from an appropriate population base so that it closely represented the target/reference population under investigation?	Y	NA	NA	Y	Y	Y	Y	Y	Y	Y
Was the selection process likely to select subjects/participants representative of the target/reference population under investigation?	Y	NA	NA	Y	Y	Y	Y	Y	Y	Y
Were measures undertaken to address and categorize non-responders?	Y	NA	NA	Y	Y	Y	Y	Y	Y	Y
Were the risk factor and outcome variables measured appropriate to the study?	Y	NA	NA	Y	Y	Y	Y	Y	Y	Y
Is it clear what was used to determine statistical significance and/or precision estimates? (e.g. p-values, confidence intervals)										
Were the methods (including statistical methods) sufficiently described to enable them to be repeated?	Y	Y	Y	Y	Y	Y	Y	Y	Y	Y
Results										
Were the basic data adequately described?	Y	Y	Y	Y	Y	Y	Y	Y	Y	Y
Does the response rate raise concerns about non-response bias?	Y	NA	NA	Y	Y	Y	Y	Y	Y	Y
If appropriate, was information about non-responders described?	Y	NA	NA	Y	Y	Y	Y	Y	Y	Y
Were the results internally consistent?	Y	Y	Y	Y	Y	Y	Y	Y	Y	Y
Were the results presented for all the analyses described in the methods?	Y	Y	Y	Y	Y	Y	Y	Y	Y	Y
Discussion										
Were the authors' discussions and conclusions justified by the results?	Y	Y	Y	Y	Y	Y	Y	Y	Y	Y

Study Characteristics

Table 3 shows a summary of the authors, interventions, outcomes, and results that were extracted from the included studies [1,3,4,10,11,19,20,22-24].

**Table 3:** Study characteristics and study outcomes. KB, Kardia Band; ECG, electrocardiographic signals; AF, atrial fibrillation; SAFE, Screening for Atrial Fibrillation; MCT, mobile cardiac telemetry; CI, confidence interval; vs, versus; LVEF, left ventricular ejection fraction; WCD, wearable cardioverter defibrillator; VT, ventricular tachycardia; ICM, implantable cardiac monitors;  AFSW, atrial fibrillation sensing watch; PPG, photoplethysmography.

Author	Journal	Arms of Study	N	Intervention Device	Condition of Interest	Follow-up Period	Outcome	Results
Bumgarner et al. (2018)	American College of Cardiology	Single arm	100	Kardia Band's (KB) smartwatch algorithm	Differentiate sinus rhythm (SR) from atrial fibrillation (AF).	3 months	Sensitivity, specificity, and K coefficient.	When the KB is supported by physician review it accurately differentiated AF from sinus rhythm. Compared to physician-interpreted ECGs, the KB demonstrated 93% sensitivity, 84% specificity, and a K coefficient of 0.77. Physician-reviewed KB recordings showed 99% sensitivity, 83% specificity, and a K coefficient of 0.83.
Cheung et al. (2018)	Canadian Journal of Cardiology	Single arm	_	Wearable technology (AliveCor Kardia Band)	Detecting arrythmias	_	Heart rate monitoring accuracy and arrhythmia detection	There is a rapid growth in the field of wearable health technologies. Heart rate monitoring: Chest strap monitors correlate highly with exercise ECGs, wrist-worn monitors, and Apple Watches. Arrhythmia detection: PPG demonstrates a 93% true positive rate. The AliveCor Kardia Band detects atrial fibrillation with 83% specificity and 93% sensitivity.
Dagher et al. (2020)	Heart rhythm journal	Single arm	_	Wearable in cardiology	Detecting and preventing CVs, especially arrhythmias	_	Expanding role of wearables in CV medicine and their potential to monitor AF continuously.	There is growth in wearable technology adoption, especially in AF detection using PPG. Potential benefits: Real-time AF management and monitoring and implications for stroke prevention. Screening for Atrial Fibrillation in the Elderly (SAFE): 1.4% of the population was detected with AF. Swedish STROKE-STOP using handheld ECG (2 weeks) identified 3% of the population with AF.
Nelson and Allen (2019)	JMIR Publications	One case report	1	Apple Watch 3 and Fitbit Charge 2.	Heart rate accuracy	2 days	Heart rate accuracy of 2 popular wearable devices	Comparing Apple Watch 3 and Fitbit Charge 2 to an ambulatory electrocardiogram (ECG) as the gold standard. Apple Watch 3: MD -1.80 bpm, 5.86% mean absolute error, and a 95% mean agreement with the ECG. Fit bit Charge 2: MD -3.47 bpm, 5.96% absolute error, and a 91% mean agreement with ECG. Both wearables showed acceptable accuracy across the 24-hour period and various activities, except for the Apple Watch 3 during activities of daily living.
Prasitlumkum et al. (2021)	Archives of Cardiovascular Diseases	Single arm	17131	Smartphones and Smartwatches	Asymptomatic atrial fibrillation (AF)	_	Accuracy of detection/diagnosis	The devices demonstrated excellent accuracy. Smartphones had a sensitivity of 94% and a 96% specificity. Smartwatches showed 94% specificity and 93% sensitivity. Wearables have a potential in early AF detection.
Sana et al. (2020)	Journal of the American College of Cardiology	Single arm	174	Wearable devices (Zio patch and NUVANT Mobile Cardiac Telemetry (MCT))	Cardiac monitoring for arrhythmias	14 days	Effectiveness of the Zio patch in detecting arrhythmias	Zio patch was found to have an overall diagnostic yield of 63.2% in detecting arrhythmias. Around 48% of the patients recorded more than 1 arrhythmia. The median time for detection of the first arrhythmia was found to be 1.0 day: 10% were symptomatic. The diagnostic yield was 63.2%. Apple's iWatch had sensitivity (87%) and specificity (97%) in silent AF identification.
Steinhubl et al. (2018)	JAMA Network	RCT	1291	Self-applied continuous ECG patch	Atrial fibrillation (AF)	4 months	Incidences of new AF diagnosis. New AF diagnosis after 1 year. Healthcare utilization. New anticoagulant prescriptions.	The study explored the effectiveness of a self-applied wearable ECG patch for detecting AF in high-risk individuals. An immediate increase in AF diagnosis after 4 months was detected compared to delayed monitoring: Immediate group (53/1366) vs. delayed group (12/1293). Monitored individuals showed increased AF diagnoses (109 vs. 81), greater initiation of anticoagulants, and higher healthcare resource utilization at 1 year: There is an association observed between active monitoring and increased anticoagulants initiation (5.7 vs 3.7 per 100 people). There was a potential for wearable ECG monitoring for early AF detection.
Tscholl et al. (2021)	ESC Heart Failure	Single arm	259	Wearable cardioverter defibrillator (WCD)	Malignant arrhythmias	2009-2017	Cardiovascular outcomes during follow-up	This study is conducted to detect arrhythmia using WCD in 259 patients with reduced ejection fraction (<50%) after myocarditis between 2009 and 2017. WCD detected sustained ventricular arrhythmias in 3%, highlighting its role in protecting high-risk patients. Findings support deferring implantable cardioverter defibrillator (ICD) placement post-myocarditis, emphasizing WCD's utility in arrhythmia detection and sudden cardiac death prevention.
Wasserlauf et al. (2019)	Journal of the American Heart Association	Single arm	24	AF-sensing watch (AFSW; Apple Watch with Kardia Band)	Atrial fibrillation (AF)	May 2017-September 2017	The primary outcome was the sensitivity of AFSW for AF episodes that were 1 hour or more. The secondary outcome was the sensitivity of the AFSW and sensitivity for total AF duration in AF detection.	The study analyzed 24 patients' simultaneous AF and AFSW recordings, 82 AF episodes ≥1 hour were detected by the implanted cardiac monitor (ICM), while the AFSW captured 80 episodes (sensitivity 97.5%). The AFSW showed high sensitivity (97.7%) in assessing AF duration. The positive predictive value for AF episodes was 39.9%.
Zhang et al. (2019)	JMIR Publications	RCT	361	PPG-based smart devices	Atrial fibrillation (AF)	14 days	Detection of atrial fibrillation	This study explores the feasibility of PPG-based smart devices for AF detection. Involving 361 subjects, the devices showed >91% predictive ability, with 100% sensitivity and approximately 99% specificity. The algorithm identified AF episodes promptly, but detection time varied based on AF type. PPG-based smart devices demonstrate potential for real-world AF monitoring, which could impact arrhythmia type on detection and monitoring duration.

Discussion

Increased use of wearable technology among the population and as recommended by physicians has had an explosive effect. Wearable devices, as observed in Table 2, have become reliable and accurate in their data collection and the management of chronic diseases such as cardiac arrhythmia. For example, the PPG smart wearable watches reported a 91% predictability of AF [23]. The smart devices also reported a 100% sensitivity and 99% specificity in detecting persistent AF. Such studies show the significant accuracies developed to help in the tests and monitoring of arrhythmias. Such milestones in technology give chances for self-monitoring for arrhythmias and other CVD. 

Research findings demonstrate a notable enhancement of 43±15% (p < 0.01) in individuals utilizing a wearable cardio defibrillator (WCD) due to their reduced left ventricular ejection fraction (LVEF) [22]. Furthermore, a recovery rate of 35% was observed among those employing WCD devices. Notably, the association between patients and physicians experienced a decline, correlating with a decrease in the average number of clinical follow-ups, ranging from 24 ± 22 months to 83 months [22]. This was an indication of how effective WCD devices are. Studies identify that hospitalization for people with emergency and life-threatening cases was 1.3 per 100 patient-years for the participants who started using wearable devices immediately after the study began to 1.4 per 100 patient-years (95% CI, -0.1, 0) [24]. Such reports show the significance of the use of wearable devices in the detection of arrhythmias [1,3,4,11,19-24].

The management of AF lies heavily in the prevention of stroke. In this case, monitoring patients was associated with the increased initiation of anticoagulants by 5.7 vs. 3.7 patients per 100 patient-years for those monitored actively and those who were not monitored [25]. Primary stroke prevention therapies have been vitamin K and direct oral coagulants. However, the development of left atrial appendage occluding devices has become an alternative therapy. With the accuracy being reported in their prediction of arrhythmias and their monitoring of heart rates, wearable devices have become an efficient means to personalize health care [26]. The outcomes indicated by the study indicated that research could be done using wearable technology. This was due to the level of accuracy observed in their monitoring. Wearable cardiac monitoring devices hold significant promise in reducing the occurrence of stroke through their ability to constantly monitor physiological parameters. This facilitates the early detection of cardiac issues from real-time data of the vital signs and cardiac risk factors supplied by the devices [27]. Recent research (2022) has shown that wearable monitoring devices are non-inferior to standard care in detecting arrhythmia. Wearable ECG devices have demonstrated considerable symptom-rhythm correlation in multiple settings, resulting in reduced time to diagnosis and thus lower rates of emergency department visits [28].

PPG technology reported a 93% true positive rate and a 54% true negative rate in detecting life-threatening arrhythmias [1]. Such accuracy could be used in studying different dimensions of heart monitoring activities due to the flexibility and portability of the devices. Studies show that the relationship between patients and physicians has helped reduce clinical visits. However, physicians may have an increased workload due to the need for interpretation, analysis, and consultation from the data collected from patients [29]. The workload is increased as the monitoring is constant and does not have to be within an immediate location. Personalization of health and treatment in preventing and managing arrhythmias becomes possible due to the portability of smart wearable devices. However, limitations may be observed due to the high costs incurred in their purchase and use. The detection of AF costs between 130 and 300 Canadian dollars (CAD) [1]. Stroke prevention costs range between 3000 and 12,000 CAD, and every AF diagnosis may cost up to 13,800 CAD.

## Conclusions

This review aimed to establish the effects of wearable technology on cardiac arrhythmias concerning their impact on the personalization of cardiac management, and also exploring their refining effect on stroke prevention strategies and their influence on research. Finally, the review discussed their preventive care of cardiac arrhythmias, and their re-evaluation of the patient-physician relationship has been covered. The study identified that wearable devices' portability promoted cardiac management personalization. Furthermore, due to their accuracy in predicting arrhythmias and heart rate monitoring, smart wearable devices were significant in the prevention of stroke and cardiac arrhythmias.

Furthermore, the use of smart wearable devices helped in advancing research in different dimensions and capacity that the researchers sought. Clinical follow-ups reduced to 24 ± 22 months up to 83 months meant that patient-physician relationships had been reduced. This gave a chance to promote the promotion of personalization of cardiac management strategies. Ultimately, using smart wearable devices to detect cardiac arrhythmias is significant and very impactful.
